# Supplementary material for: Epidermal Loss of RORα Enhances Skin Inflammation in a MC903-Induced Mouse Model of Atopic Dermatitis
Source: Int J Mol Sci. 2023 Jun 16;24(12):10241. doi: 10.3390/ijms241210241 (PMC10298918; doi:10.3390/ijms241210241)
Supplement: Supplementary file 1 [file ijms-24-10241-s001.zip › ijms-2380968-supplementary.pdf]

SUPPLEMENTARY INFORMATION

Epidermal loss of RORa enhances skin inflammation in MC903-induced mouse model of atopic dermatitis

Xiangmei Hua,[1] Conrad Dean Blosch,[2] Hannah Dorsey,[1] Maria K. Ficaro,[1] Nicole L. Wallace,[1] Richard P. Hsung,[1] Jun Dai [1,3]*

[1] School of Pharmacy, University of Wisconsin-Madison, WI, USA

[2] Biomedical Research Model Services, University of Wisconsin-Madison, WI, USA

[3] UW Carbone Cancer Center, University of Wisconsin-Madison, WI, USA

* Corresponding author:

Jun Dai, PhD

Assistant Professor

The School of Pharmacy

University of Wisconsin, Madison

777 Highland Avenue

Madison, WI 53705

Tel: 608-292-3196

Email: jdai32@wisc.edu

Supplementary Figure S1

A. Figure 1C

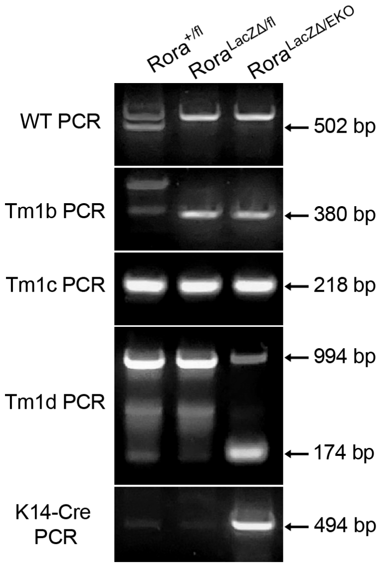

B.

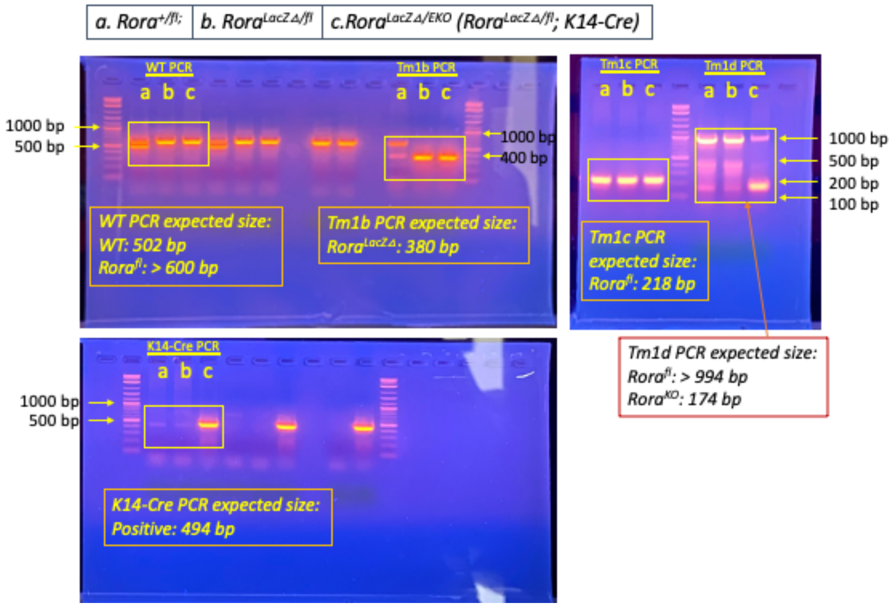

Figure S1. Validation of epidermal *Rora* depletion in *Rora*$^{LacZ\Delta/EKO}$ mice using short-range end-point PCR assays. (**A**) Genotyping results are shown in **Figure 1C** in the main text. (**B**) Original DNA gel images for the images are shown in (**A**). The PCR products were separated by 2% agarose gels and stained with ethidium bromide.

Supplementary Figure S2

A. Figure 2A

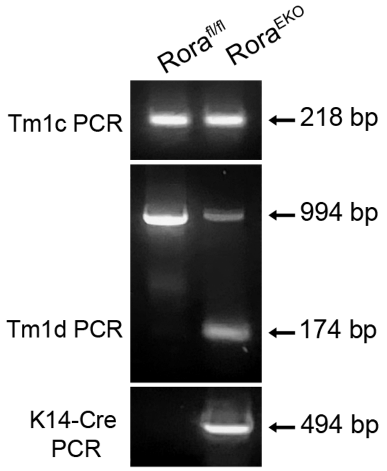

B.

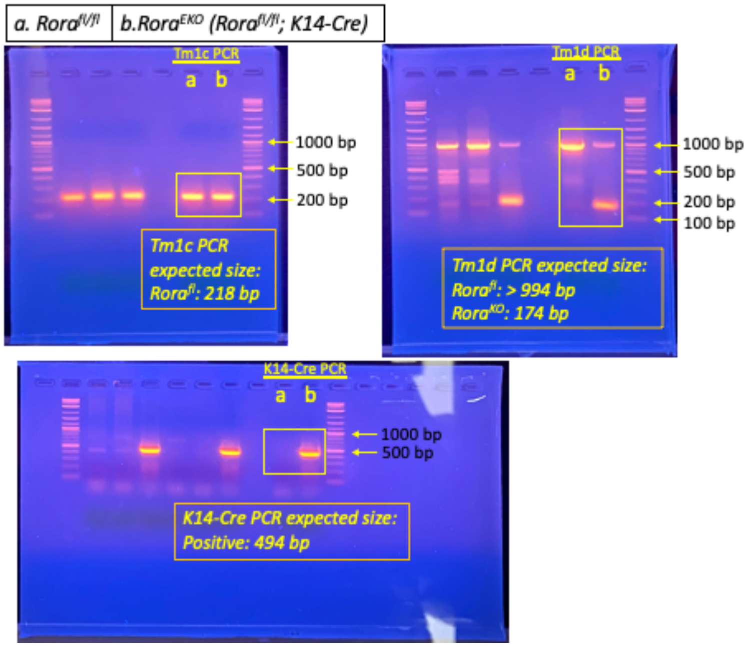

Figure S2. Validation of epidermal *Rora* depletion in *Rora*^{EKO} mice using short-range end-point PCR assays. (**A**) Genotyping results are shown in **Figure 2A** in the main text. (**B**) Original DNA gel images for the images are shown in (**A**). The PCR products were separated by 2% agarose gels and stained with ethidium bromide.

Supplementary Figure S3

A. Figure 6C

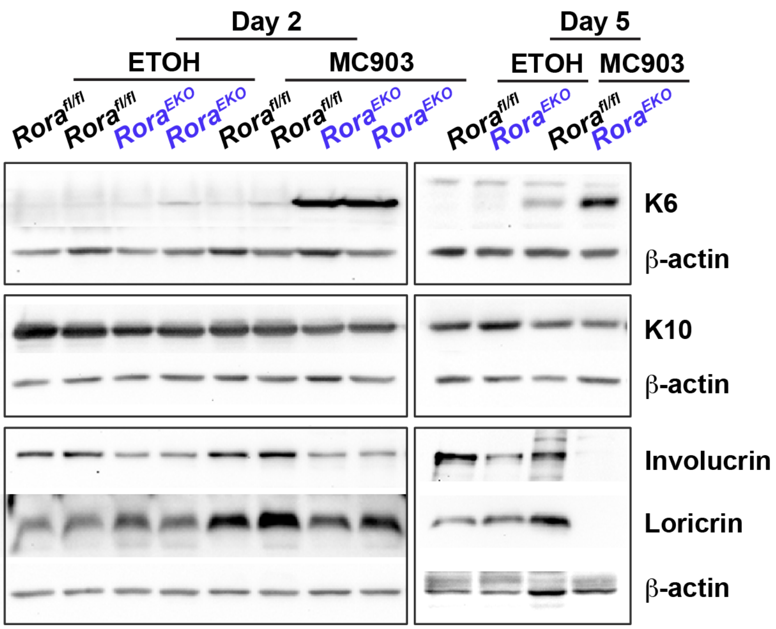

B.

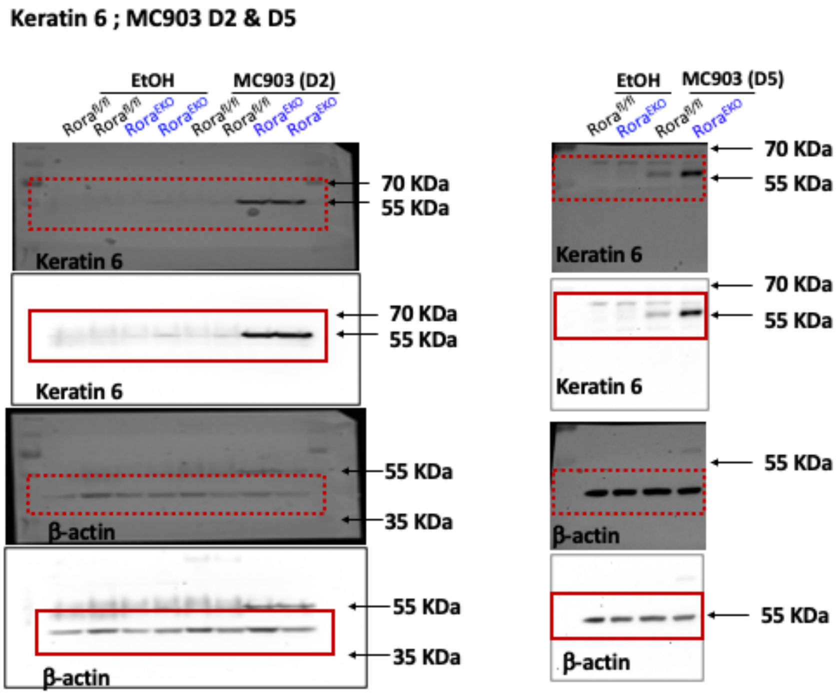

C.

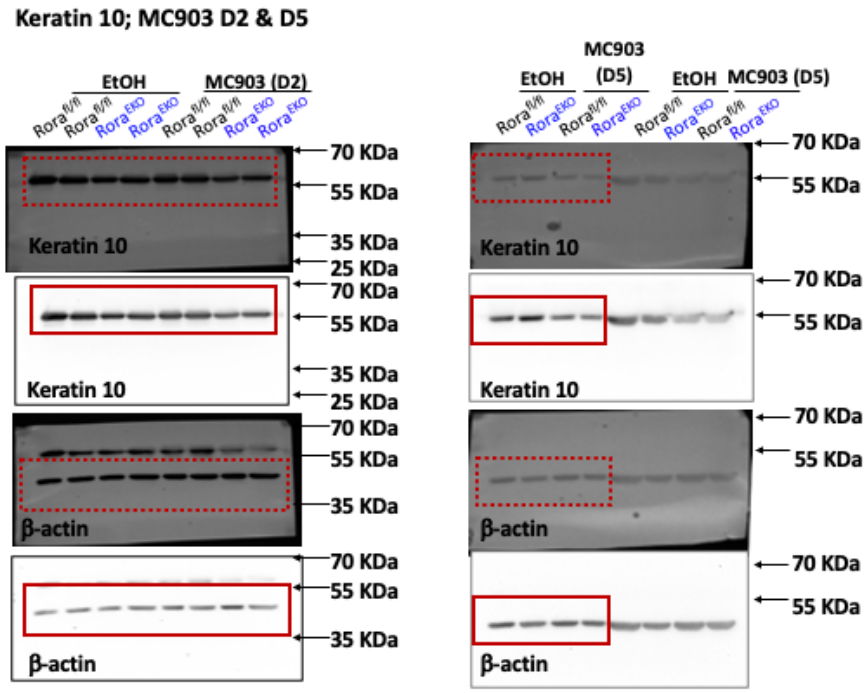
Keratin 10; MC903 D2 & D5
EtOH
MC903 (D2)
MC903 (D5)
EtOH
MC903 (D5)
Rora fl/fl
Rora EKO
70 KDa
55 KDa
35 KDa
25 KDa
Keratin 10
β-actin

D.

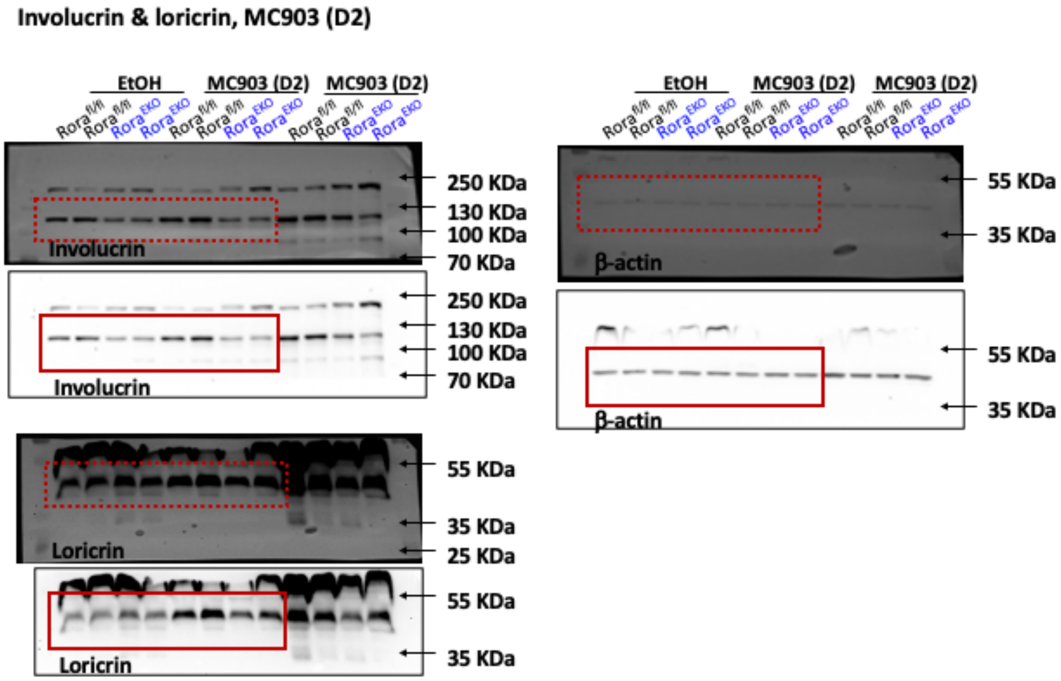
Involucrin & loricrin, MC903 (D2)
EtOH
MC903 (D2)
MC903 (D2)
Rora fl/fl
Rora EKO
250 KDa
130 KDa
100 KDa
70 KDa
55 KDa
35 KDa
25 KDa
Involucrin
Loricrin
β-actin

E.

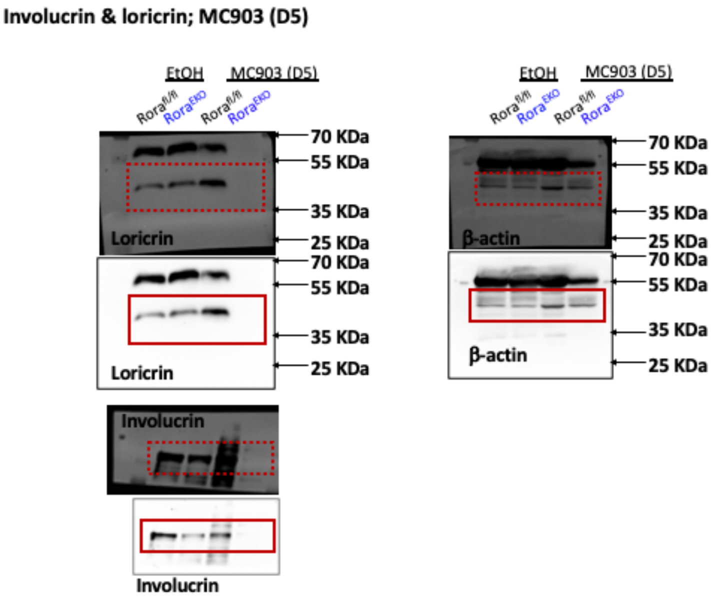

F.

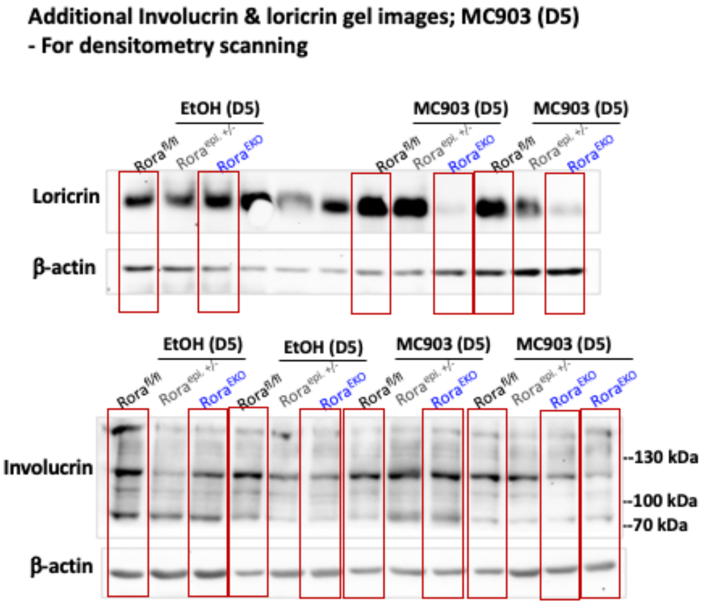

Figure S3. Western blot analysis of indicated proteins in ear samples collected on day 2 after EtOH- or MC903-treatment. (**A**) Western blot analysis results are shown in **Figure 6C** in the main text. (**B-E**) Original protein gel images for the images are shown in (**A**). For each band, the top image shows the membrane overlay and the lower image shows the chemiluminescence signal. **(F)** Additional gel images used for protein quantification shown in Figure 6D.

Supplementary Table S1

Supplementary Table S1: Primer sequences of mouse genes for RT-PCR and genotyping		
**Genes**		
**Primers for RT-PCR Analysis**	**Forward**	**Reverse**
Rora	CTCGCTAGAGGTGGTGTTTATT	GTCTTCACAACCTAGGGACTTG
Il1b	GGCAGGCAGTATCACTCATT	GAGGATGGGCTCTTCTTCAAA
Cxcl2	ATCCAGAGCTTGAGTGTGACGC	AAGGCAAACTTTTTGACCGCC
Cxcl10	GAATCCGGAATCTAAGACCATCAA	GTGCGTGGCTTCACTCCAGT
Krt16	GGTGGCCTCTAACAGTGATCT	TGCATACAGTATCTGCCTTTGG
Tslp	GCTACCCTGAAACTGAGAGAAA	TCTGGAGATTGCATGAAGGAATA
Sprr2a	GCCTTGTCGTCCTGTCATGT	GGCATTGCTCATAGCACACTAC
Cyp24a1	CTGCCCCATTGACAAAAGGC	CTCACCGTCGGTCATCAGC
**PCR primers for genotyping**	**Forward**	**Reverse**
Rora WT	GAGAAGCTGCTGATCACTACA	CTCTTGTGTATACCACCACA
Tm1b	CGGTCGCTACCATTACCAGT	ACTGATGGCGAGCTCAGACC
Tm1c	AAGGCGCATAACGATACCAC	CCGCCTACTGCGACTATAGAGA
Tm1d	AAGGCGCATAACGATACCAC	ACTGATGGCGAGCTCAGACC
K14-CRE	TTCCTCAGGAGTGTCTTCGC	GTCCATGTCCTTCCTGAAGC

Supplementary Table S2

Supplementary Table S2: Primary antibodies used for immunostaining and western blot		
**Name**	**Source**	**Catalog no.**
Loricrin	BioLegend (Covance), Dedham, MA	pRB-145P
F4/80-Biotin	Bio-Rad, Hercules, CA	MCA497
Keratin 10	BioLegend (Covance)	PRB-159P
Keratin 6	BioLegend (Covance)	PRB-169P
TSLP	R&D Systems, Minneapolis, MN	AF555
CD4	BioLegend (Covance)	100446
CD11c	BioLegend (Covance)	117346
β-Actin (13E5)	Cell Signaling Technology, Danvers, MA	4970S
